# The effects of flexible short protocol with gonadotropin-releasing hormone antagonist on preventing premature ovulation in poor responders

**DOI:** 10.1007/s00404-023-07287-z

**Published:** 2023-12-05

**Authors:** Yan Zhang, Hongyou Wang, Xinyue Zhang, Yingying Hao, Jihong Yang, Yangbai Li, Ting Feng, Yandong Chen, Yun Qian

**Affiliations:** 1 Reproductive Center, Second Affiliated Hospital, Nanjing Medical University, Nanjing, 210011 China; 2 Department of Obstetrics and Gynecology, Binhai County People’s Hospital, Yancheng, 224000 China

**Keywords:** Poor ovarian response, Ovarian stimulation, Premature ovulation, GnRH antagonist, Pregnancy rate, Live birth

## Abstract

**Purpose:**

The proportion of patients with poor ovarian response (POR) is increasing, but effective treatment remains a challenge. To control the hidden peaks of luteinizing hormone (LH) and premature ovulation for poor responders, this study investigated the efficacy of flexible short protocol (FSP) with gonadotropin-releasing hormone antagonist (GnRH-ant) on trigger day.

**Methods:**

The 662 cycles of POR patients were retrospectively analyzed. The cohort was divided into control and intervention groups. The intervention group (group A) with 169 cycles received a GnRH-ant given on trigger day. The control (group B) with 493 cycles received only FSP. The clinical outcomes of the two groups were compared.

**Results:**

Compared with group B, with gonadotropin-releasing hormone antagonist (GnRH-ant) on trigger day in group A the incidences of spontaneous premature ovulation decreased significantly (2.37% vs. 8.72%, *P* < 0.05). The number of fresh embryo-transfer cycles was 45 in group A and 117 in group B. There were no significant differences in clinical outcomes, including implantation rate, clinical pregnancy rate, live birth rate and the cumulative live birth rate (12.0% vs. 9.34%; 22.22% vs. 21.93%; 17.78% vs. 14.91%; 20.51% vs. 20%, respectively; *P* > 0.05) between the two group.

**Conclusion:**

FSP with GnRH-ant addition on trigger day had no effect on clinical outcomes, but could effectively inhibit the hidden peaks of luteinizing hormone (LH) and spontaneous premature ovulation in POR. Therefore, it is an advantageous option for POR women.

**Supplementary Information:**

The online version contains supplementary material available at 10.1007/s00404-023-07287-z.

## What does this study add to the clinical work

**Table:** 

In clinical practice, reproductive doctors need flexibility to treat POR patients. FSP with GnRH-ant addition on trigger day may be a potential option to control the hidden peaks of luteinizing hormone (LH) and premature ovulation with no effect on clinical outcomes.	

## Introduction

As the prevalence of infertility continues to rise, more patients seek the help of assisted reproductive technology (ART). Although the development of ART has solved many fertility problems, patients with poor ovarian response (POR) still face significant challenges. Based on the Bologna consensus [1, 2], POR is diagnosed if two of the following three criteria are met: (a) advanced age (≥ 40 years) or other risk factors for adverse ovarian reaction; (b) number of oocytes retrieved by routine induction of ovulation cycles in previous cycle ≤ 3; (c) abnormal ovarian reserve (antral follicle count [AFC] < 5–7 or anti-Müllerian hormone [AMH] < 0.5–1.1 ng/ml). The incidence of POR is about 9–24% [3], and the pregnancy rate in POR patients is about 10–20% [4], significantly lower than in the normal population.

Reproductive clinicians improve oocyte quality and clinical outcome of POR patients by adding growth hormone (GH) [5], recombinant luteinizing hormone (LH) (rLH) [6–8], dehydroepiandrosterone (DHEA) [9], and coenzyme Q10 (CoQ10) [10] during controlled ovarian stimulation (COS). Many strategies for the treatment of POR, including micro-stimulation protocol, ultra-short regimen, short regimen, antagonist regimen, and natural cycle, have also been compared. The use of gonadotropin-releasing hormone (GnRH) agonists or antagonists has been extensively analyzed, but without conclusive results [11, 12]. The natural cycle or mini-stimulation in vitro fertilization (IVF) has been reported to be a patient-friendly option [13–15], and showed that the implantation rate was significantly higher with natural cycles than with shorter GnRH agonist regiments [16]. However, a high cancellation rate due to premature ovulation remains a disadvantage. In clinical practice, POR patients often need to undergo multiple IVF cycles. In repeated IVF treatments, the incidence of POR tends to increase, the treatment period gets prolonged, and the economic burden increases [17]. These might bring great psychological pressure to both doctors and patients. As a result, selecting an appropriate ovulation induction program for POR patients is critical.

In patients with POR, LH spontaneous surges often appear as hidden peaks; therefore, it is difficult to control spontaneous premature ovulation, which is also one of the main reasons for the high cancellation rate. Gonadotropin-releasing hormone antagonist (GnRH-ant) can rapidly inhibit LH secretion [18], thus preventing premature luteinization and ovulation in COS [19, 20]. Some studies showed that GnRH-ant may affect follicle growth and endometrial receptivity [21]. Compared with the antagonist regimen, the gonadotropin-releasing hormone agonist regimen is more beneficial to improve the clinical pregnancy rate and live birth rate [22]. Flexible short protocol (FSP) is a "delayed start" protocol for gonadotropins, and the initial stage of cyclic follicle recruitment and dominant follicle selection could be carried out before the addition of exogenous gonadotropins. Our previous studies have shown that flexible short protocol (FSP) has an advantage over traditional short and mild stimulus programs in POR women over 40 years of age [23], which increases oocyte and embryo quality and achieves a higher clinical pregnancy rate. Researchers had improved their approach by combining GnRH-ant with natural cycles or gonadotropin agonist cycles [13, 24, 25]. In this study, according to clinical work experience, GnRH-ant was added to POR patients on the trigger day of FSP treatment, to observe its effectiveness in reducing premature ovulation rate and evaluate the safety of pregnancy outcomes.

## Materials and methods

### Study population

A retrospective analysis was conducted on data extracted from clinical records of patients with POR who underwent IVF/intracytoplasmic injection (ICSI) treatment in the Reproductive Medicine Centre of the Second Affiliated Hospital of Nanjing Medical University from January 2016 to December 2020. The study was approved by the hospital ethics committee.

*Inclusion criteria*: (i) FSP ovulation induction cycle; (ii) patients with POR.

Patients who added GnRH-ant on the trigger day were classified as Group A (the intervention group, with GnRH-ant addition group), while those who did not add were classified as Group B (the control group, without GnRH-ant addition group). Both groups A and B used the FSP protocol.

*Exclusion criteria*: (i) Hyperprolactinemia or combined with thyroid, adrenal, and other endocrine system diseases; (ii) intrauterine adhesion, uterine space occupying lesions or congenital uterine malformation; (iii) chromosomal abnormalities; (iv) surgical sperm extraction, frozen sperm, donor sperm, etc.

### Ovarian stimulation protocols

#### FSP

Triptorelin (0.03 mg/d or 0.05 mg/d, Ferring AG, Switzerland) was administered from day 3 of the menstrual cycle until the previous day of HCG-day, and gonadotropin (follicle-stimulating hormone [FSH], 150U, Lizhu, China) was initiated when oestradiol (E2) began to rise, and at least one follicle had grown to 5 mm in diameter. Follicular development was monitored by ultrasound every other day during this period. The timing of gonadotropin injection was more flexible, starting from the 5th to 10th day of menstruation. When more than 50% of the dominant follicle had a ≥ 16 mm diameter, HCG (6500U, Lizhu, China) was injected intramuscularly at about 20:00 and/or GnRH-ant (0.5 mg, Merck Sharp & Dohme, America) was injected subcutaneously in the day.

### Oocyte collection, fertilization, and embryo culture

**Fig. 1:**
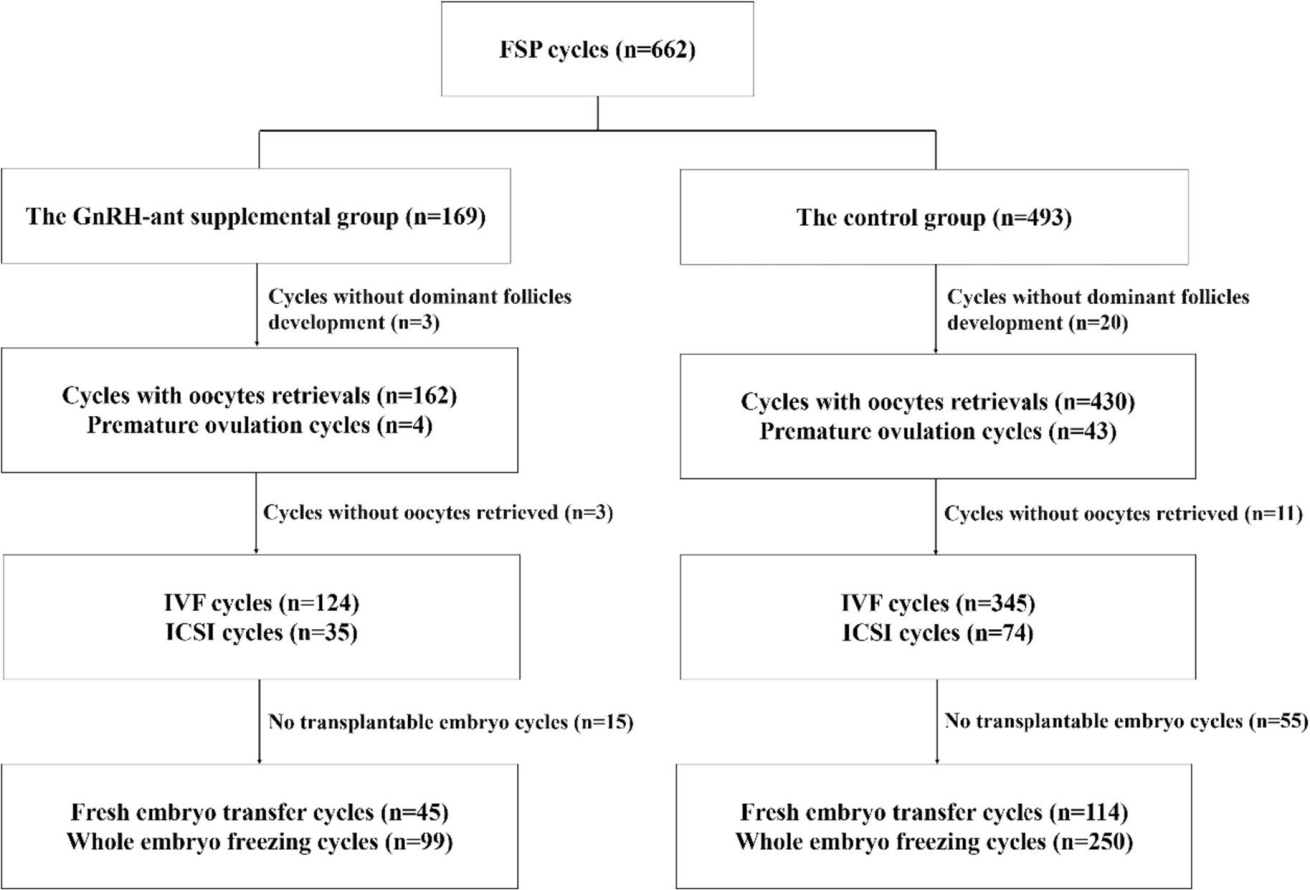
The study flowchart. *FSP* flexible short protocol, *GnRH* gonadotropin-releasing hormone, *ICSI* intracytoplasmic sperm injection, *IVF* in vitro fertilization

The cumulus–oocyte complexes (COCs) were absorbed by vaginal aspiration 36 h after the HCG injection. Based on semen analysis, oocytes were fertilized either by IVF or ICSI. Embryo quality had been assessed daily based on the Istanbul Consensus Workshop on Embryo Assessment [26]. On the third day, transplantable embryos were transplanted and cultured blastocysts for vitrification freezing (Fig. 1).

### Fresh cycle transfer cancellation

Cancellation was based on five criteria: (i) P ≥ 1.5 ng/ mL on trigger day; (ii) embryo factors including no oocytes, no normal fertilization, or no embryo transfer (ET), etc.; (iii) endometrial factors including endometrial polyps, endometritis, etc.; (iv) some patients choose to accumulate embryos (in subsequent cycles embryos from two cycles may be transplanted at the same time); (v) miscellaneous factors including patient discomfort on the proposed transplant day, the partner's absence, incomplete documents, etc.

### Embryo transfer and luteal support

Ultrasound-guided fresh ET was performed on day 3 with a maximum of two embryos transferred. Endometrium preparation was performed using a hormone replacement cycle for frozen-embryo transfer. Luteal support was followed by oral progesterone (P, Abbott, America) at 40 mg/d. After 14 days, either hCG-positive urine or blood hCG ≥ 50mIU/ml established the diagnosis of biochemical pregnancy. Clinical pregnancy was defined as the presence of a gestational sac with or without fetal heart activity under ultrasound examination 4 weeks after embryo transfer.

### Outcome assessment

The main outcome measures were premature ovulation rate, clinical pregnancy rate, live birth rate, and cumulative live birth rate. The assessment of premature ovulation was before the oocyte retrieval. Premature ovulation is the release of the dominant follicle prior to oocyte retrieval. Clinical pregnancy was defined as the presence of a gestational sac with or without fetal heart activity under ultrasound examination 4 weeks after embryo transfer. Miscarriage rate was defined as the proportion of patients experiencing spontaneous pregnancy loss before 12 weeks of gestational age. Live birth was defined as a delivery of a live neonate at ≥ 28 weeks of gestation. Clinical pregnancy and live birth rate per patient were defined as number of clinical pregnancies and live births divided by the number of patients. Cumulative live birth rate was calculated as the first live birth from all fresh and frozen–thawed embryos transferred after the oocyte retrieval of patients with fresh embryo-transfer cycles.

### Statistical analysis

SPSS 26.0 statistical software was used for data analysis and processing. The data were expressed by means ± standard deviation ($\overline{x }$ ± s). *T* test and nonparametric test were used for normal and non-normal distributions, respectively. Chi-square test was used to compare the rate (%) between the two groups. *P* < 0.05 was considered statistically significant.

## Results

### Baseline characteristics

A total of 662 FSP cycles of 349 POR women were included in this study. The intervention group (group A, 169 cycles) and control group (group B, 493 cycles) were divided according to whether GnRH-ant (0.5 mg) was injected on the trigger day. Patient ages ranged from 23 to 53 years, with an average of 40.68 ± 5.60 years in group A, and 40.57 ± 6.66 years in group B. Between the two groups, duration of infertility, FSH, LH, E2, AMH, and AFC had no significant differences. The results are shown in Table 1.

**Table 1:** Baseline characteristics of the Group A and B

	Group A	Group B	*P*
Number of cycles	169	493	NA
Age (years)	40.68 ± 5.60	40.57 ± 6.66	0.827
Duration of infertility (years)	4.83 ± 4.37	4.07 ± 3.63	0.051
BMI (kg/m^2^)	22.44 ± 3.41	22.92 ± 2.64	0.585
Day 2–4 FSH (mIU/ml)	11.64 ± 5.19	12.62 ± 9.23	0.872
Day 2–4 LH (mIU/ml)	4.15 ± 2.44	5.21 ± 7.85	0.059
Day 2–4 E2 (pg/ml)	69.58 ± 64.54	71.60 ± 68.62	0.939
AMH (ng/ml)	0.75 ± 0.68	0.63 ± 0.66	0.137
AFC	5.96 ± 2.35	5.63 ± 2.80	0.162
Length of stimulation (days)	8.42 ± 3.45	8.59 ± 4.04	0.949
Total dose of gonadotropin used (IU)	1837.28 ± 967.65	1762.56 ± 1027.29	0.277
Serum E2 (pg/ml) on the trigger day	1359.43 ± 1256.77	1130.45 ± 1118.68	0.009*
Serum LH (mIU/ml) on the trigger day	4.53 ± 2.76	5.12 ± 7.07	0.368
Serum P (ng/ml) on the trigger day	1.06 ± 1.34	1.00 ± 0.98	0.835
Premature ovulation rate (%)	2.37% (4/169)	8.72% (43/493)	0.006*
Number of cycles with oocytes retrievals	162	430	NA
Number of oocytes retrieved	2.81 ± 2.17	2.57 ± 2.03	0.068

Group A: the intervention group (with GnRH-ant addition group); Group B: the control group (without GnRH-ant addition group)

Data is expressed as mean ± SD, or number (percentage). Independent t test. Nonparametric test. Chi-squared test. *P < 0.05

Premature ovulation rate (%) = number of follicular premature ovulation cycles/number of ovulation cycles

*AFC* antral follicle count, *AMH* anti-Müllerian hormone, *E2* oestradiol, *FSH* follicle-stimulating hormone, *LH* luteinizing hormone, *P* progesterone, *NA* not applicable

### Association between the addition of GnRH-ant, oocyte development, and embryo quality

The average dose and duration of gonadotropin use in group A and B were similar (1837.28 ± 967.65 vs. 1762.56 ± 1027.29; 8.42 ± 3.45 vs. 8.59 ± 4.04). On trigger day, serum LH and P were showed no significant differences between the two groups. The premature ovulation rate of group A was 2.37%, which was significantly lower than in group B (8.72%; *P* < 0.05). These results suggest that GnRH-ant supplementation can effectively prevent the occurrence of premature ovulation. The number of cycles with oocytes retrievals in group A was 162, and 430 in group B. Although serum E2 was higher in group A, the number of oocytes retrieved did not increase significantly (Table 1).

IVF and ICSI cycles of group A were 124 and 35, and those of group B were 345 and 74, respectively (*P* > 0.05, Table 2, Supplementary Table 2). No differences were noted between the two groups in normal fertilization rate and high-quality embryo rate. The number of transplantable embryos and transplantable embryos rate in group A were lower than in group B, suggesting that the addition of GnRH-ant may affect embryo development. The freezing rate of whole embryos in group A was 58.58%, which was higher than in group B (50.71%); however, there was no statistical difference.

**Table 2:** Laboratory characteristics of Group A and B

	Group A	Group B	*P*
Insemination modes	159	419	0.232
IVF cycles	77.99% (124/159)	82.34% (345/419)	
ICSI cycles	22.01% (35/159)	17.66% (74/419)	
Average number of transplantable embryos	1.65 ± 1.49	1.70 ± 1.29	0.723
Transplantable embryo rate (%)	82.57% (270/327)	88.04% (685/778)	0.015*
High-quality embryo rate (%)	59.02% (193/327)	65.04% (506/778)	0.058
Whole embryo freezing rate (%)	58.69% (99/169)	50.71% (250/493)	0.077

Group A: the intervention group (with GnRH-ant addition group); Group B: the control group (without GnRH-ant addition group)

Data is expressed as mean ± SD, or number (percentage). Independent t test. Nonparametric test. Chi-squared test. *P < 0.05

Transplantable embryo rate (%) = number of transplantable embryos/number of 2PN; high-quality embryo rate = number of high-quality embryos (grade I and II embryos)/number of 2PN; whole embryo freezing rate (%) = number of whole embryos freezing cycles/number of oocytes retrieved cycles

*ICSI* intracytoplasmic sperm injection, *IVF* in vitro fertilization, *NA* not applicable

### Effect of GnRH-ant addition on clinical outcomes

In Table 3, the transfer cycle of the fresh ET cycle was 45 cycles in group A and 117 cycles in group B. The average number of embryos transferred was 1.67 ± 0.64 (group A) and 1.60 ± 0.57 (group B); the difference was not statistically significant. The clinical pregnancy rate in group A was 22.22%, similar to that in group B. The implantation, miscarriage, and live birth rates in group A were 12.0%, 20.0%, and 17.78%, respectively, which were not significantly different from group B (9.34%, 32%, and 14.91%, respectively). Between group A and group B, there was no difference in the clinical pregnancy and live birth rate per patient (25.64% vs. 25%; 20.51% vs. 17%, respectively; *P* > 0.05). During the frozen embryo-transfer cycles, there were no live birth in group A and 3 live births in group B. In cumulative live birth rate, the difference was also not statistically significant (20.51% vs. 20%; *P* > 0.05). The results indicated that GnRH-ant addition on trigger day did not affect the pregnancy outcomes.

**Table 3:** Pregnancy outcomes of Group A and B

	Group A	Group B	*P*
Fresh embryo-transfer cycles	45	114	NA
Mean of embryos transferred	1.67 ± 0.64	1.60 ± 0.57	0.680
Implantation rate (%)	12.0% (9/75)	9.34% (17/182)	0.520
Clinical pregnancy rate (%)	22.22% (10/45)	21.93% (25/114)	0.968
Clinical pregnancy rate per patient (%)	25.64% (10/39)	25% (25/100)	0.938
Miscarriage rate (%)	20% (2/10)	32% (8/25)	0.478
Live birth rate (%)	17.78% (8/45)	14.91% (17/114)	0.655
Live birth rate per patient (%)	20.51% (8/39)	17% (17/100)	0.628
Cumulative live birth rate (%)	20.51% (8/39)	20% (20/100)	0.946

Group A: the intervention group (with GnRH-ant addition group); Group B: the control group (without GnRH-ant addition group)

Data is expressed as mean ± SD, or number (percentage). Independent t test. Nonparametric test. Chi-squared test. *P < 0.05

Implantation rate (%) = number of clinically pregnant embryos/number of total embryos transferred. Clinical pregnancy rate (%) = number of clinical pregnancy cycles/total transplant cycles; miscarriage rate (%) = number of miscarriage cycles/number of clinical pregnancy cycles; live birth rate (%) = number of live birth cycles/total transplant cycles; clinical pregnancy rate per patient (%) = number of clinical pregnancy cycles/number of patient; live birth rate per patient (%) = number of live birth cycles/number of patient; cumulative Live birth rate (%) = number of first live birth cycles (all fresh and frozen embryo-transfer cycles)/number of patient

*NA* not applicable

In addition, according to the Poseidon criteria [27, 28], our analysis found that more than 70% of cycles fell into the group 4 (patients ≥ 35 years, AFC < 5, and AMH < 1.2 ng/ml). Although the number of cycles from group 1 to group 3 was too small to be statistically comparable, the results of the group 4 were consistent with the Bologna consensus, as detailed in the Supplementary Table 1. These once again demonstrated the effectiveness and safety of our protocol.

## Discussion

In this trial, we evaluated the efficacy of FSP with GnRH-ant addition in reducing premature ovulation rates in patients with POR. The results showed that the GnRH-ant addition could control ovulation of dominant follicles well, and was beneficial for in vitro fertilization. In terms of the prognosis, the similar pregnancy outcomes suggested no adverse effects on endometrial receptivity and embryo implantation. Therefore, FSP with GnRH-ant addition can prevent premature ovulation without affecting the clinical outcomes.

The risk factors for premature ovulation in patients with POR remain unclear. GnRH-ant administration has been reported to rapidly and profoundly inhibit the secretion of endogenous LH [29]. GnRH-ant binds to the GnRH receptor in the pituitary gland to effectively inhibit LH secretion, premature luteinization, and ovulation. And a higher central GnRH tone may be present in patients with poor prognosis. The current preferred dose of GnRH-ant in ART therapy is 0.25 mg [30, 31]. Studies have shown that the current dosage does not prevent breakthrough ovulation in normal women, which may be attributed to a reduction in plasma concentration of GnRH-ant [32]. In patients using GnRH-ant who have a history of breakthrough ovulation, double use of GnRH-ant in subsequent cycles has been recommended [32, 33]. GnRH-ant has a half-life of 30 h after subcutaneous injection and a plasma concentration duration of more than 20 h [34]. In this study, the time from injection of GnRH-ant to oocyte collection was more than 40 h. Based on these, a single injection of 0.5 mg of GnRH-ant was used, which was higher than the usual dose but more convenient than daily injections. Results showed that it could effectively inhibit follicular premature ovulation.

Although the GnRH-ant regimen could significantly reduce the incidence of ovarian hyperstimulation syndrome (OHSS), the GnRH agonist regimen was beneficial for improving pregnancy rate and live birth rate [22, 35, 36]. GnRH-ant administration may have negative effects on follicular growth, oocyte development, and endometrium receptivity, and it may interfere with the embryo implantation window and luteal function [21, 37–39]. Moreover, patients who discontinued GnRH-ant on trigger day showed significant improvement in embryonic outcomes [40]. Based on these concerns, we compared the outcomes of the two groups of patients with or without GnRH-ant supplementation. Our findings showed that there was no significant difference in the number of oocytes retrieved, but in group A, the transplantable embryo rates were lower than in group B. Results suggest that the addition of GnRH-ant may affect embryo development. However, in the fresh ET cycle, the clinical pregnancy, implantation, and live birth rates were similar between the two groups. There was also no statistical difference in the cumulative live birth rates between group B and group A, indicating that the injection of GnRH-ant on trigger day had no effect on pregnancy outcomes in POR.

We analyzed the reason why GnRH-ant supplementation had no effect on clinical outcomes. First, it has also been reported that low LH does not affect the prognosis of POR patients [41] and the difference in the rate of transferable embryos is an individual bias of the population. Second, as mentioned in the literature [40], the slight fluctuation of LH level before ovulation has little influence on the quality of oocytes in patients with POR. The duration of the single injection was shorter than that of the antagonist protocol, and follicle development was mature on the trigger day. Hence, the effect on prognosis is small. Finally, the average number of transplantable embryos in both groups was less than 2 embryos in patients with POR. Many patients had only one opportunity for transfer. Differences in the transplantable embryo rate had no significant effect on clinical outcomes in patients with POR.

Clinicians had modified various regimens to try to find one that will universally benefit all patients with POR. Our study showed that a single addition of antagonist on trigger day is effective for POR. Compared to traditional and modified natural cycles, patients could have reduced cycle cancellation rates, access to embryo transfer opportunities, and satisfactory pregnancy rates; to related combination regiments such as GnRH agonist/GnRH-ant, our regiments are single- and low-dose antagonists that are more beneficial to patients [7, 8, 13, 24, 42–44]. GnRH-ant regimens had been showed that implantation rates and pregnancy rates decreased compared to agonists regimens, which had negative effects on endometrial receptivity during the fresh cycle [45–47]. P-primed ovarian stimulation (PPOS) protocols also achieved ovulation control of dominant follicles [48], but this protocol could not achieve fresh cycle embryo transfer. In our plan, approximately 50% of patients could choose fresh embryo transfer. Fresh cycle transplantation can save patients' time and cost, and reduce the long-term impact of frozen-embryo transfer [49].

Of course, this study has some limitations that need to be acknowledged. First, this study is the author's clinical work to analyze the advantages of using antagonist addition in the treatment of POR, but the exact mechanism still needs basic experimental research. Second, it was a retrospective cohort study wherein the number of patients with POR recruited in each group was different. The sample size of fresh ET was limited, which may have affected clinical outcomes. Third, the dose of GnRH-ant was 0.5 mg, without comparing 0.25 mg and other doses. Thus, randomized-controlled trials with a larger sample size are needed to further validate our observations.

## Conclusion

The FSP with GnRH-ant addition on trigger day can prevent premature ovulation without affecting pregnancy outcomes. The single administration of GnRH-ant injection seemed to be more convenient for patients and did not carry the risk of long-term LH inhibition. This method is effective and safe, and may be a potential option for POR patients.

### Supplementary Information

Below is the link to the electronic supplementary material.

Supplementary material 1 (.docx 22 KB)

## Data Availability

The data supporting the results of this study can be obtained from the corresponding authors upon reasonable request.

## Abbreviations

AFC: Antral follicle count
AMH: Anti-Müllerian hormone
ART: Assisted reproductive technology
COC: Cumulus-oocyte complex
COS: Controlled ovarian stimulation
GH: Growth hormone
DHEA: Dehydroepiandrosterone
CoQ10: Coenzyme Q10
ET: Embryo transfer
E2: Oestradiol
FSH: Follicle-stimulating hormone
FSP: Flexible short protocol
GnRH: Gonadotropin-releasing hormone
hCG: Human chorionic gonadotropin
ICSI: Intracytoplasmic sperm injection
IVF: In vitro fertilization
LH: Luteinizing hormone
OHSS: Ovarian hyperstimulation syndrome
P: Progesterone
POR: Poor ovarian response
