# Supplementary material for: The effects of flexible short protocol with gonadotropin-releasing hormone antagonist on preventing premature ovulation in poor responders
Source: Arch Gynecol Obstet. 2023 Dec 5;309(2):689–97. doi: 10.1007/s00404-023-07287-z (PMC10808440; doi:10.1007/s00404-023-07287-z)
Supplement: Supplementary file 1 — Supplementary material 1 (.docx 22 KB) [file 404_2023_7287_MOESM1_ESM.docx]

**Supplementary Table 1** Clinical outcomes of Group A and B with Poseidon concept.

|  | Group A | Group B | p |
| --- | --- | --- | --- |
| Number of cycles  Group -1  Group -2  Group -3  Group -4 | 169  8  21  15  125 | 493  10  46  93  344 | NA  NA  NA  NA  NA |
| Premature ovulation rate (%)  Group -1  Group -2  Group -3  Group -4 | 2.37%（4/169）  0%（0/8）  9.52%（2/21）  0%（0/15）  1.6%（2/125） | 8.72%（43/493）  10%（1/10）  4.35%（2/46）  4.30%（4/93）  10.47%（36/344） | 0.006*  0.357  0.407  0.413  0.002* |
| Fresh embryo-transfer cycles  Group -1  Group -2  Group -3  Group -4 | 45  3  6  5  31 | 114  2  13  27  72 | NA  NA  NA  NA  NA |
| Clinical pregnancy rate (%)  Group -1  Group -2  Group -3  Group -4 | 22.22% (10/45)  66.67%（2/3）  16.67%（1/6）  40%（2/5）  16.13%（5/31） | 21.93% (25/114)  0%（0/2）  30.77%（4/13）  33.33%（9/27）  16.67%（12/72） | 0.968  0.136  0.516  0.773  0.946 |

Group A: the intervention group (with GnRH-ant addition group); Group B: the control group (without GnRH-ant addition group).

Data is expressed as mean ± SD, or number (percentage). Chi-squared test.

* P < 0.05. NA: not applicable.

**Supplementary Table 2** Clinical outcomes of IVF and ICSI in Group A and B

|  | Group A | Group B | p |
| --- | --- | --- | --- |
| Insemination modes | 159 | 419 | 0.232 |
| IVF cycles | 124 | 345 | NA |
| ICSI cycles | 35 | 74 | NA |
| Fresh embryo-transfer cycles | 45 | 114 | 0.867 |
| IVF cycles | 37 | 95 | NA |
| ICSI cycles | 8 | 19 | NA |
| Clinical pregnancy rate (%) | 22.22% (10/45) | 21.93% (25/114) | 0.968 |
| IVF cycles | 24.32% (9/37) | 22.11% (21/95) | 0.665 |
| ICSI cycles | 12.50% (1/8) | 21.05% (4/19) | 0.601 |
| Live birth rate (%) | 17.78% (8/45) | 14.91% (17/114) | 0.655 |
| IVF cycles | 18.92% (7/37) | 14.74% (14/95) | 0.555 |
| ICSI cycles | 12.50% (1/8) | 15.79% (3/19) | 0.826 |

Group A: the intervention group (with GnRH-ant addition group); Group B: the control group (without GnRH-ant addition group).

Data is expressed as mean ± SD, or number (percentage). Chi-squared test.

* P < 0.05. NA: not applicable.
